# Transcriptome Analysis of the Vernalization Response in Barley (*Hordeum vulgare*) Seedlings

**DOI:** 10.1371/journal.pone.0017900

**Published:** 2011-03-09

**Authors:** Aaron G. Greenup, Sharyar Sasani, Sandra N. Oliver, Sally A. Walford, Anthony A. Millar, Ben Trevaskis

**Affiliations:** 1 Division of Plant Industry, CSIRO, Canberra, Australian Capital Territory, Australia; 2 Research School of Biology, Australian National University, Canberra, Australian Capital Territory, Australia; 3 Department of Cereals Research, Seed and Plant Improvement Institute, Karaj, Tehran, Iran; University of Massachusetts Amherst, United States of America

## Abstract

Temperate cereals, such as wheat (*Triticum spp.*) and barley (*Hordeum vulgare*), respond to prolonged cold by becoming more tolerant of freezing (cold acclimation) and by becoming competent to flower (vernalization). These responses occur concomitantly during winter, but vernalization continues to influence development during spring. Previous studies identified *VERNALIZATION1* (*VRN1*) as a master regulator of the vernalization response in cereals. The extent to which other genes contribute to this process is unclear. In this study the Barley1 Affymetrix chip was used to assay gene expression in barley seedlings during short or prolonged cold treatment. Gene expression was also assayed in the leaves of plants after prolonged cold treatment, in order to identify genes that show lasting responses to prolonged cold, which might contribute to vernalization-induced flowering. Many genes showed altered expression in response to short or prolonged cold treatment, but these responses differed markedly. A limited number of genes showed lasting responses to prolonged cold treatment. These include genes known to be regulated by vernalization, such as *VRN1* and *ODDSOC2*, and also contigs encoding a calcium binding protein, 23-KD jasmonate induced proteins, an RNase S-like protein, a PR17d secretory protein and a serine acetyltransferase. Some contigs that were up-regulated by short term cold also showed lasting changes in expression after prolonged cold treatment. These include *COLD REGULATED 14B* (*COR14B*) and the barley homologue of *WHEAT COLD SPECIFIC 19* (*WSC19*), which were expressed at elevated levels after prolonged cold. Conversely, two *C-REPEAT BINDING FACTOR* (*CBF*) genes showed reduced expression after prolonged cold. Overall, these data show that a limited number of barley genes exhibit lasting changes in expression after prolonged cold treatment, highlighting the central role of *VRN1* in the vernalization response in cereals.

## Introduction

In temperate regions, wheat (*Triticum spp.*) and barley (*Hordeum vulgare*) can be sown in autumn to grow vegetatively through winter before flowering in spring. Autumn sowing can enhance yield relative to later sowing times, but can also expose plants to freezing winter conditions [1]. Consequently, the capacity to survive winter frosts is an important trait for autumn-sown wheat and barley varieties grown in regions that experience cold winters [2]–[4].

Tolerance to winter frosts is established through cold acclimation, the process where freezing tolerance increases as temperatures decrease during autumn [5]. Molecular analyses have identified low-temperature responsive genes that are induced during cold acclimation, such as ice crystallisation inhibitors and dehydrins, which protect against freezing damage [5]. *C-REPEAT BINDING FACTOR* (*CBF*) genes encode transcription factors that play a critical role in the cold acclimation process [5]. *CBF* genes are rapidly induced by low temperatures to activate genes that contribute to increased freezing tolerance [5]. The *FROST RESISTANCE 2* (*FR2*) locus on chromosome 5A of wheat and barley (5H), which is associated with variation in frost tolerance [6]–[8], has been mapped to a cluster of *CBF* genes [9]–[12], reviewed in [13]. An increased number of *CBF* genes at the *FR2* locus might enhance cold acclimation in frost tolerant varieties [14].

Another feature of many autumn-sown wheats and barleys is the vernalization requirement; the requirement for prolonged exposure to cold to make plants competent to flower. The requirement for vernalization delays reproductive growth and stem elongation before winter, minimising the risk of frost damage to cold-sensitive reproductive organs. Furthermore, since the capacity for cold acclimation decreases during reproductive growth, the requirement for vernalization allows more time for cold acclimation by lengthening the vegetative growth phase [15]. Consistent with this hypothesis, varieties sown in autumn in regions that experience cold winters typically have a strong requirement for vernalization and can acclimate to cold over an extended period. In comparison, varieties that flower without vernalization have a shorter vegetative growth phase and less time for cold acclimation, and are consequently less frost tolerant [3], [4], [15].

During winter, low temperatures overcome the vernalization requirement and trigger a quantitative flowering response, so that longer exposure to cold causes more rapid flowering until the vernalization response is saturated after several weeks at low-temperatures [16]–[19]. The initial response to prolonged cold can be separated from the flowering response; for example, when sprouting seeds are vernalized, inflorescence development (flowering) does not begin until plants are shifted to normal growth temperatures [17], [20]. This implies a memory of cold that mediates a quantitative change in the rate of development after vernalization.

In cereals, vernalization-induced flowering is mediated by the activation of *VERNALIZATION1* (*VRN1*), a gene that promotes flowering (reviewed in [21], [22]). *VRN1* transcript levels show a quantitative response to cold, with longer durations of cold activating *VRN1* expression to greater extents, and expression of *VRN1* is maintained when vernalized plants are shifted to normal growth temperatures [23]–[27]. This long term activation of *VRN1* might be mediated through vernalization-induced changes in the state of chromatin at the *VRN1* locus [28].

Following vernalization, *VRN1* accelerates flowering by promoting the transition to reproductive development at the shoot apex and by making the leaves competent to respond to increasing daylength during spring, which accelerates inflorescence development and stem elongation [17], [20], [29]. Many varieties of wheat and barley carry alleles of *VRN1* that are expressed at elevated levels without prior cold treatment [23]–[27], [23]–[27]. These varieties flower without vernalization and can be grown in warm climates, where winter temperatures are not cold enough for vernalization, or sown in spring.

By accelerating the transition to reproductive growth, when cold acclimation is inhibited, active alleles of *VRN1* are likely to limit the potential for cold acclimation and reduce frost tolerance [15]. Consistent with this hypothesis, *VRN1* is linked to the *FR1* locus, which influences the activity of cold acclimation pathways and frost tolerance [6], [7], [9], [23]–[27]. Furthermore, deletion of the *VRN1* region increases the activity of cold acclimation pathways, and frost tolerance, in the *maintained vegetative phase mutant* (*mvp*) of *Triticum monococcum* [37], which grows vegetatively indefinitely [38]. These observations suggest that *VRN1* and *FR1* are likely the same gene [37]. The impact of the *VRN1* deletion or active *VRN1* alleles on frost tolerance is greatest in long-days, where floral development occurs rapidly [15], [37].

Cold acclimation and vernalization occur concomitantly when autumn-sown plants experience low-temperatures during winter, but vernalization continues to influence development when plants return to warm temperatures. In this study we use microarrays to compare and contrast gene expression in seedlings of a vernalization-responsive barley (cv Sonja) exposed to short or prolonged cold treatments. Additionally, we compare gene expression in the leaves of vernalized versus non-vernalized plants. By comparing the effects of short and prolonged cold on the transcriptome we identify genes that show distinct low temperature responses. We were also able to identify genes that show lasting responses to prolonged cold treatment, an expression pattern that defines vernalization-responsive genes. Possible roles for these genes in the vernalization response of cereals are discussed.

## Results

**Figure 1:**
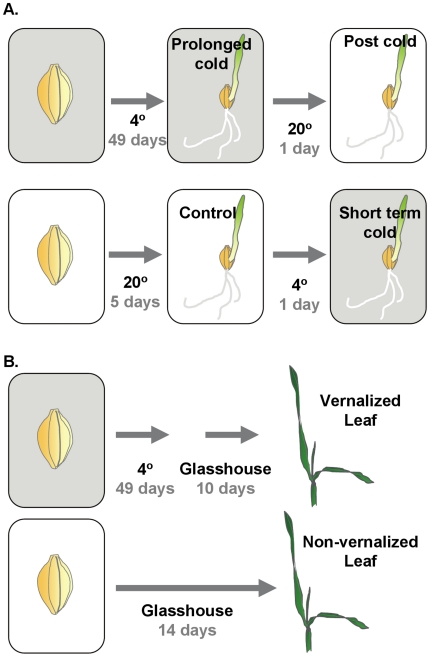
Overview of sampling methods for microarray analysis. A) Barley seeds (cv. Sonja) were germinated and grown in darkness at either 20°C over 5 days (control) or 4°C over 49 days (prolonged cold). Seedlings were then shifted from the control treatment to 4°C for 24 hours (short term cold) or shifted from the prolonged cold treatment to 20°C for 24 hours (post cold). In all treatments the shoot apex remained at an early stage of vegetative development, but plants grown from seedlings that experienced prolonged cold flower rapidly when shifted to normal growth conditions, unlike control seedlings germinated at 20°C [20]. B) To identify contigs that show a sustained response to prolonged cold, barley seeds were germinated in the dark at 4°C for 49 days and then transferred to growth in glasshouse conditions until they reached the three leaf stage (10 days after the end of cold treatment). Non-vernalized control plants were grown simultaneously under the same conditions and were sampled at the equivalent developmental leaf stage (14 days). Grey shading indicates low-temperature.

**Figure 2:**
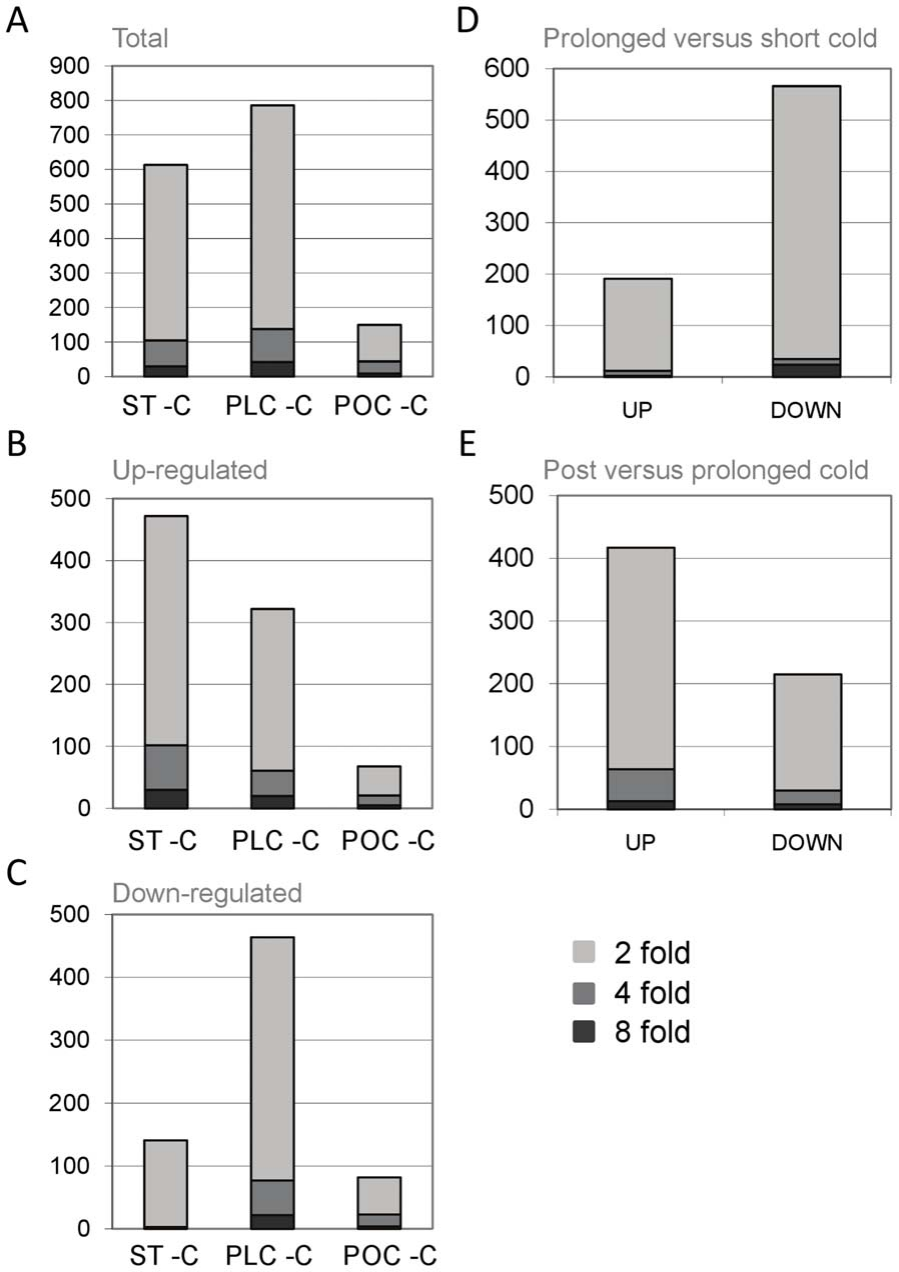
Changes in gene expression in seedlings after different low-temperature treatments. A) The total numbers of contigs showing differential expression (>2 fold change, p<0.01) in the short term cold versus control (ST-C), the prolonged cold versus control (PLC-C) or the post cold versus control (POC-C) samples. B) The total numbers of genes up-regulated in the same comparisons. C) Genes down-regulated in the same comparisons. D) The total number of contigs up or down-regulated in the prolonged versus short term cold treatments. E) The number of contigs up or down-regulated in the post versus prolonged cold treatments. Dark shading indicates 8 fold change of greater, intermediate shading indicates 4–8 fold, light shading indicates 2–4 fold change.

**Figure 3:**
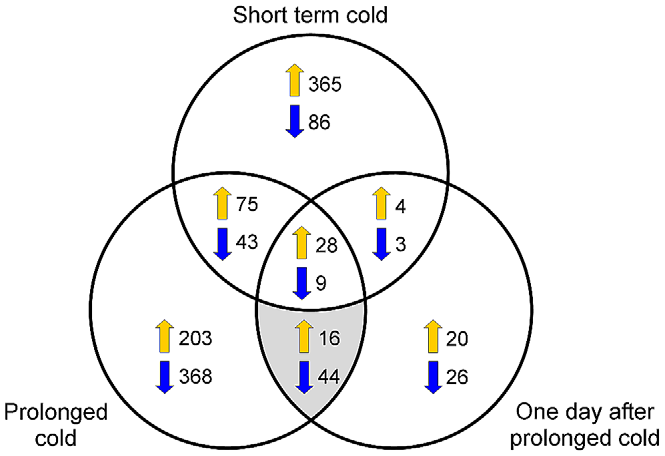
Venn diagram showing the differentially expressed contigs across the different treatments. A summary of the contigs that showed a two fold or greater change in transcript levels across the different treatments when compared to the control treatment (p<0.1). Shaded area indicates contigs that were significantly changed in the samples treated with prolonged cold and one day after prolonged cold treatment, relative to the control.

The Affymetrix 22K Barley1 chip [39] was used to examine the effects of short or prolonged cold on the transcriptome of barley seedlings (see materials and methods, Figure 1A). Pair wise comparisons of the different treatments were used to identify contigs that have altered expression in the short term, prolonged or post-cold treatments (Tables S1, S2, S3, S4, S5, S6). Large numbers of contigs show altered expression (> two fold change relative to the non-treated control, p<0.01) in short (613 contigs, Table S1) or prolonged (786 contigs, Table S2) cold treatment (Figure 2). The contigs that showed altered expression after short term cold were predominantly up-regulated, whereas many contigs were down-regulated in the prolonged cold treatment (Figure 2). Fewer contigs showed altered expression in the post cold treatment relative to the non-treated control (150 contigs, Table S3). A limited number of contigs showed a lasting response to prolonged cold; i.e. significantly changed in both the prolonged cold and 1 day after prolonged cold samples, but not the short term cold sample (60 contigs) (Figure 3). Expression levels were verified for a subset of contigs with contrasting expression patterns, using quantitative RT-PCR (Figure S1).

**Figure 4:**
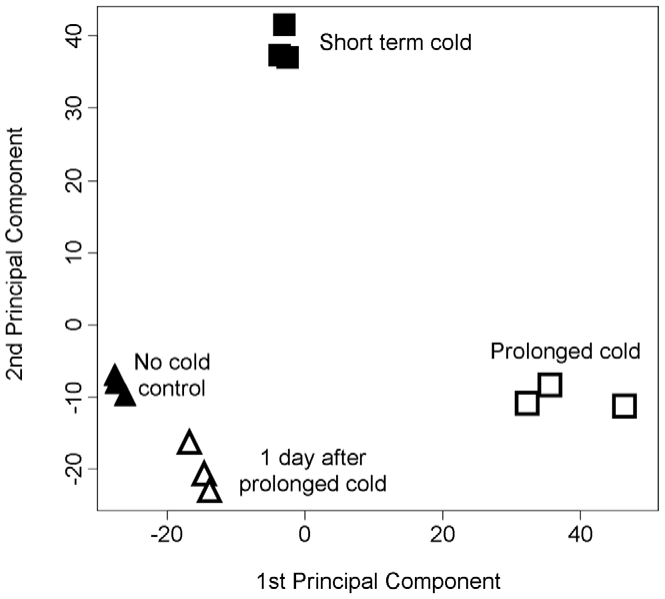
Principle component analysis of microarray data. Principal component analysis was applied to differentially expressed contigs identified in the different seedling treatments (see methods). Closed triangles (▴) indicate samples from the no cold control treatment. Open triangles (**Δ**) represent the 1 day after prolonged cold treatment. Closed squares (▪) represent the short term cold treatment. Open squares (□) represent the prolonged cold treatment.

Principal component analysis (PCA) [40] was used to visualise the overall changes in gene expression in the different treatments (Figure 4). The first two principal components, which explained 97% of the total variance (95% 1^st^ component, 1.8% second component), show that the short term and prolonged cold treatments were dissimilar to each other and to the control treatment (Figure 4). In comparison the post cold treatment was more similar to the control treatment than to either the short or prolonged cold treatments. Replicates showed a high degree of similarity for all four treatments (Figure 4). The results of PCA analysis are consistent with the number of contigs that show altered expression in each treatment, relative to the control (Figures 2, 3, Table S1, S2, S3, S4, S5, S6). Taken together, these observations indicate that prolonged cold treatment has a strong influence on the transcriptome of barley, distinct to that of short term cold treatment, but the majority of cold-induced alterations to the transcriptome are not maintained when plants are shifted to warm conditions after prolonged cold treatment.

### K-means cluster analysis

**Figure 5:**
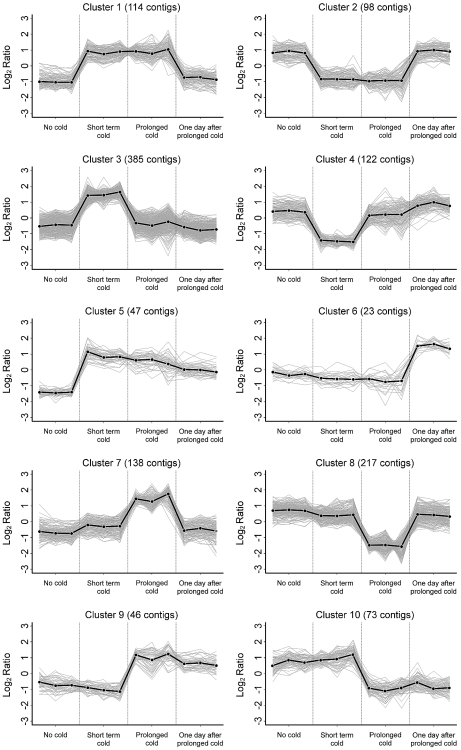
K-means cluster analysis of differentially expressed contigs. The mean for each cluster is shown as black dots and lines and the gray lines represent the expression pattern of individual contigs. The three replicates for each treatment data are shown.

Cluster analysis was used to analyse the expression patterns of contigs that showed significant changes in expression (p<0.01) in any of the two way comparisons between seedling treatments. Ten primary clusters were identified; each showing distinctive expression patterns (Figure 5, ).

A cluster of 114 contigs showed elevated expression at low-temperature, irrespective of the duration (Figure 5, cluster 1). Contigs belonging to this cluster correspond to cold acclimation genes, such as *DEHYDRIN5* (*DHN5*) (contig1717_s_at), *DELTA1-PYRROLINE-5-CARBOXYLATE SYNTHASE 1* (*P5CS1*) (contig3814_at) and *GALACTINOL SYNTHASE* (contig3810_at and contig3811_at) (Table S7; contig sequences are available at www.plexdb.org, see Barley Annotation [41]). A second cluster of 98 contigs (cluster 2) showed an inverse expression pattern, and is made up of contigs that are down-regulated by low-temperatures (Figure 5, cluster 2). This cluster includes contigs corresponding to heat-shock genes (contig2006_s_at and contig5597_s_at) and a *PROLINE OXIDASE* (contig68_at) (Table S7).

A group of 85 contigs showed increased expression only in the short term cold treatment (Figure 5, cluster 3). This cluster includes several contigs annotated as transcription factors, examples include: basic leucine zipper domain, zinc finger domain, WRKY and NAC domain transcription factors. There were also several contigs annotated as *GLUTATHIONE-S-TRANSFERASE* genes (Table S7). Another cluster showed the inverse pattern and was made up of 122 contigs (Figure 5, cluster 4). This cluster includes contigs annotated as core histone domain containing proteins (contig175_at and contig175_x_at) and basic helix-loop-helix transcription factors (contig4559_s_at, contig4560_at, contig4560_x_at and contig26382_at) (Table S7). Clusters 3 and 4 define contigs that respond to short term cold exposure or “cold shock”.

A group of 47 contigs showed elevated expression in the short term cold treatment, the prolonged cold treatment and the post cold sample (Figure 5, cluster 5). Contigs belonging to this cluster include several contigs corresponding to cold acclimation genes (Table S7). Twenty three contigs showed elevated expression only in the post cold samples (Figure 5, cluster 6). This cluster includes heat-shock genes (contig2004_s_at and contig2007_s_at) and auxin or jasmonate responsive genes (contig17690_at and contig2906_at, HVSMEg0005M23r2_at respectively) (Table S7).

Contigs that showed elevated expression only in the prolonged cold treatment were grouped in cluster 7 (138 contigs) (Figure 5). This cluster includes contigs corresponding to *FLOWERING LOCUS T-like2* (HVSMEl0003G02r2_at) and an *APETALA2-like* gene (contig18652_at) (Table S7). Conversely, contigs that had decreased expression in prolonged cold treatment were grouped into cluster 8 (217 contigs) (Figure 5). Examples from this cluster include contigs described as zinc finger transcription factors (contig4486_at and contig8233_s_at) and cysteine proteases (HB26O11r_at, HVSMEl0003G02r2_at and contig11505_at) (Table S7).

Of most interest to the aims of this study were contigs that showed a lasting response to prolonged cold. A cluster of 46 contigs showed increased expression in the prolonged cold and post cold samples (Figure 5, cluster 9). This cluster includes *VRN1* (rbaal14f06_s_at), contigs corresponding to *23kd JASMONATE INDUCED* genes (rbags15p13_s_at and contig1679_s_at), a putative glucan synthase (contig19065_at) and a calcium binding EF-hand protein (AJ250283_at) (Table S7). Another cluster of 73 contigs showed the inverse pattern, with decreased expression in the prolonged cold and post cold samples (Figure 5, cluster 10). This group includes contigs corresponding to *ODDSOC2* (*HvOS2*) (contig12031_at), rubisco activase (contig1019_at) and RNase S-like proteins (contig5059_s_at and contig5058_x_at) (Table S7).

### Vernalization-responsive genes

Gene expression was assayed in the fully expanded second leaf of vernalized or non-vernalized plants using the Affymetrix 22K Barley1 chip [39] (see materials and methods, Figure 1B). This allowed comparisons between vernalized and non-vernalized plants to be made using developmentally equivalent tissues, which cannot be made if apex tissue is included (vegetative in non-vernalized versus reproductive in vernalized plants at this growth stage, [20]). A total of 60 contigs showed greater than two fold change in expression level (p<0.01) in vernalized versus non-vernalized leaves. A less stringent criteria identified 244 contigs that showed greater than 1.5 fold changes in expression level (p<0.05) (Table S8). Of these, 120 were up-regulated after vernalization, including contigs corresponding to *VRN1* (rbaal14f06_s_at), *COR14b* (HVSMEa0015E13r2_s_at) and a *JUMONJI* transcription factor (contig24321_at). A total of 128 contigs showed lower expression levels after vernalization, including contigs corresponding to *XYLOGLUCAN ENDOTRANSGLYCOSYLASE* (*XET*) (HVSMEb0004L16r2_at, contig2673_at and contig2670_x_at), *HvOS2* (contig12031_at) and *CBF9* (HVSMEn0019L21f_at).

**Figure 6:**
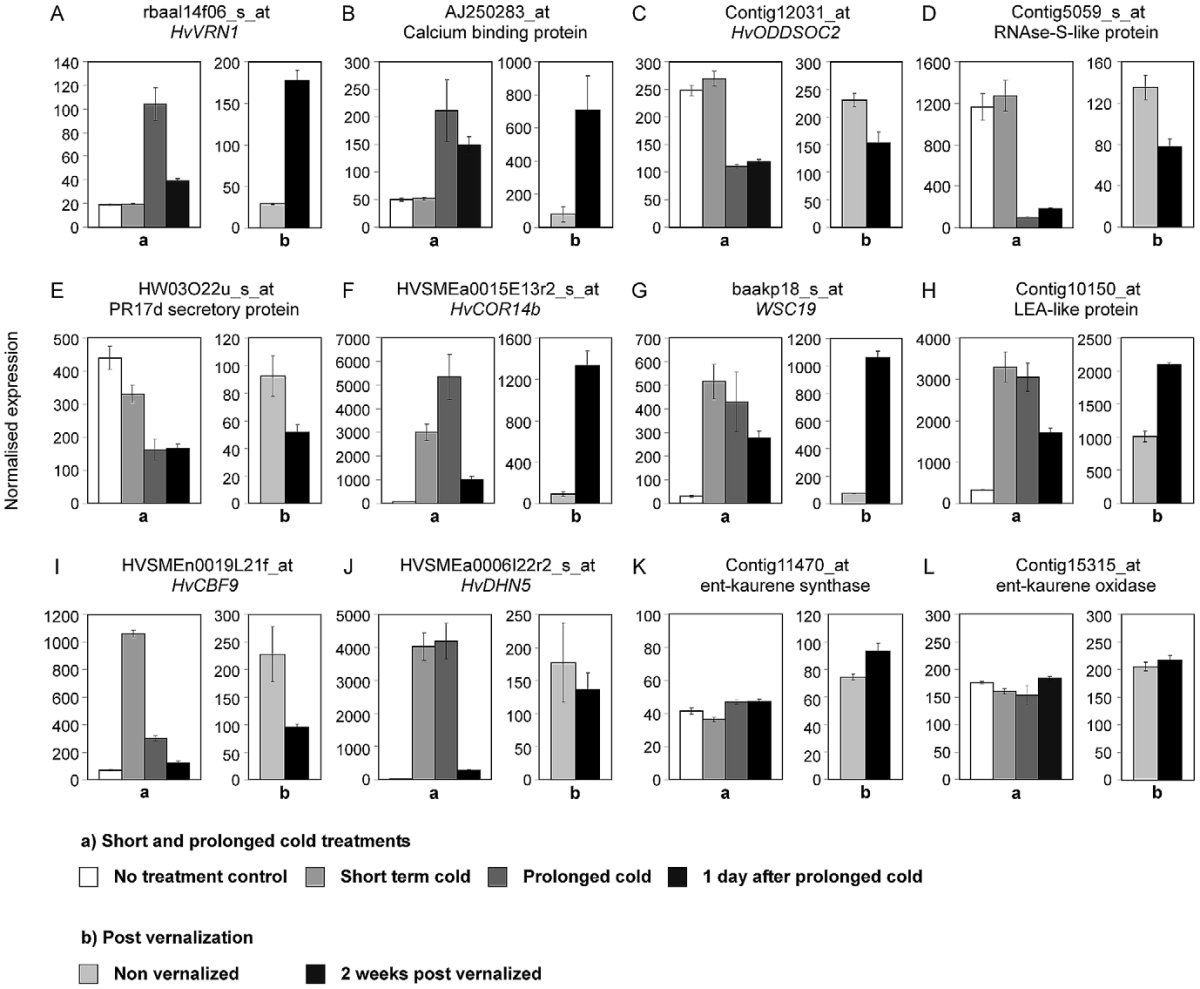
Examples of temperature responsive contigs. The normalized expression levels for individual contigs (A–L), as assayed by Affymetrix Barley1 Chip (RMA normalization) for control seedlings, short term or prolonged cold treatment, and one day after prolonged cold (a). Also shown are expression levels for contigs in the fully expanded second leaf of plants after vernalization (10 days post-vernalization), versus the equivalent leaves from non-vernalized control plants (b).

The prolonged cold treatment sample (see above) corresponds to the end of the vernalization treatment (49 days at 4°C). Of the contigs that showed altered expression in the leaves of plants after vernalization, 14 showed altered expression in both the prolonged cold and 1 day after prolonged cold seedling treatments (clusters 9 and 10) (end of vernalization treatment): six were up-regulated including *VRN1* (rbaal14f06_s_at) and a calcium binding protein (AJ250283_at) (Figure 6A, B, Table 1). In addition, eight were down-regulated including contigs for *HvOS2* (contig12031_at), RNAse-S-like protein (contig5059_s_at) and a PR17d secretory protein (HW03O22u_s_at) (Figure 6C–E, Table 1).

**Table 1:** Contigs that show a lasting response to cold in the leaves of vernalized plants.

Probe Set	Best Match	Fold Change	*P* Value
**Cluster 9**			
AJ250283_at	Calcium binding EF-hand protein	11.39	5.90E-04
rbaal14f06_s_at	*VRN1* (MADS box gene)	6.12	2.63E-09
rbags15p13_s_at	Jasmonate induced protein	5.43	1.47E-03
Contig1679_s_at	Jasmonate-induced protein	1.81	1.33E-04
Contig372_s_at	Unknown	1.70	1.69E-04
Contig19065_at	*Glucan synthase-like 3* (*Hordeum vulgare*)	1.60	7.59E-04
**Cluster 10**			
Contig12031_at	*ODDSOC2* (MADS box gene)	−1.52	3.19E-03
Contig23272_at	Serine acetyltransferase protein, putative	−1.55	1.07E-04
HS07I12u_s_at	FAD binding domain, monooxygenase	−1.58	3.72E-03
HE01O15u_at	extracellular dermal glycoprotein-like	−1.67	3.60E-04
HVSMEf0015C12f_at	unknown	−1.68	1.13E-03
Contig5059_s_at	RNase S-like protein	−1.73	5.94E-04
HW03O22u_s_at	PR17d/secretory protein	−1.75	3.07E-03
Contig9743_at	Unknown	−1.79	1.61E-03

Some contigs that respond to short term cold also had altered expression in the leaves of vernalized plants. These include *COR14b* (HVSMEa0015E13r2_s_at) (Figure 6F, Table S9), a cold acclimation protein WCS19 (baakp18_s_at) (Figure 6G, Table S9) and a LEA-like protein (contig10150_at) (Figure 6H, Table S9). Others did not show consistent expression patterns when the seedling treatments were compared to the leaf samples. *CBF9* (HVSMEn0019L21f_at) showed elevated expression after short term and prolonged cold but was down-regulated in the leaves of plants after vernalization (Figure 6I, Table S9). The expression pattern of these contigs is distinct to other contigs that are cold regulated. For example, contigs corresponding to *DHN5* (contig1717_s_at and HVSMEa0006I22r2_s_at) are also induced by cold but show no change in expression in the leaves of vernalized plants (Figure 6J). A full list of the contigs with altered expression in seedlings and leaves is provided in Table S9.

## Discussion

Previous studies investigated the effects of low-temperature on the transcriptome of wheat or barley by examining short to medium term cold responses (1 day–2 weeks) [42]–[45]. In this study, transcriptional responses to short or prolonged cold were assayed and compared (Figure 1). The data presented show that transcriptional responses to short or prolonged cold differ markedly. This is evident from the lists of contigs with significantly changed expression for each treatment relative to the control (Figure 2, Tables S1, S2, S3, S4, S5, S6) and is further highlighted by PCA (Figure 4). Comparing and contrasting the effects of different lengths of cold treatment identified contigs potentially involved in different low-temperature responses. Contigs that respond to short term cold treatment are likely to function during cold “shock”, to adjust homeostasis to rapid decreases in temperature, whereas contigs that show altered expression after prolonged cold are likely to be important for long term growth at low temperatures. Comparison of this dataset with previous microarray analyses of low-temperature responses in barley identified 55 contigs that showed a significant response to cold in all experiments (8 down and 47 up) (Table S10) (Plexdb accession no. BB65 and BB81; [42]). These contigs define a core set of low-temperature responsive genes from barley, including genes previously identified as cold responsive, such *DHN5* [43], that are likely to play critical roles in cold acclimation (HVSMEa0006I22r2_s_at and contig1717_s_at) (Table S10).

A limited number of contigs show a sustained response to prolonged cold after plants were shifted to warm conditions (Figures 2, 3, 5). Moreover, few of the contigs that showed altered expression in the prolonged cold treatment showed altered expression in the leaves of vernalized plants at the third leaf stage (10 days after the end of a prolonged cold treatment) (Table 1). This observation is important with regards to the phenomenon of vernalization-induced flowering, since contigs that show a lasting response to prolonged cold potentially contribute to the acceleration of reproductive development that occurs in vernalized plants (see [20]). Indeed, the list of contigs identified as showing a sustained response to prolonged cold includes *VRN1*, a central regulator of the vernalization response in cereals [23].

*HvOS2* was identified amongst contigs that are down-regulated by vernalization (Figure 6C, Table 1). This is consistent with previous studies, which showed that *HvOS2*, and two closely related wheat genes, *Triticum aestivum AGAMOUS-like 33* and *42* (*TaAGL33*, *TaAGL42*), show reduced transcript levels during and after vernalization [26], [46], [47]. Down-regulation of *HvOS2* in vernalized plants is likely to contribute to accelerated flowering [47], and although *HvOS2* is down-regulated by cold independently of *VRN1*, maintained repression of *HvOS2* after vernalization requires *VRN1* [47]. Thus, down-regulation of *HvOS2* in the leaves of vernalized plants can be considered a consequence of *VRN1* expression. An *RNase S-like* gene (contig5059_s_at), which is up-regulated by *HvOS2* [47], was down-regulated in the leaves of vernalized plants (Figure 6D, Table 1). Although the function of this gene is not known, this expression pattern is consistent with the hypothesis that transcription of this gene is activated by *HvOS2*.

A number of other vernalization-responsive contigs were identified. These include a contig predicted to encode a calcium binding EF-hand protein (AJ250283_at), which showed increased expression after vernalization (Figure 6B, Table 1). EF hand proteins act as calcium sensors and likely contribute to diverse biological processes, including hormone metabolism, cell signalling and gene expression (reviewed in [48]). Calcium signalling might play a role during short term cold responses and cold acclimation [45], [49], [50]. The identification of a vernalization-responsive gene encoding an EF-hand protein (contig AJ250283_at) suggests that altered calcium signalling might also play a role during post-vernalization development in cereals.

Contigs corresponding to two 23kd jasmonate induced proteins (rbags15p13_s_at and contig1679_s_at), which might regulate cell wall polysaccharide synthesis [51], were induced by vernalization, as was a contig corresponding to a glucan synthase (contig19065_at) (Table 1). Conversely, a contig corresponding to a FAD binding domain containing protein (HS07I12u_s_at) was down-regulated (Table 1). Altered transcript levels for these contigs might reflect adjustment of metabolism in vernalized plants to facilitate the transition to reproductive growth. Alternatively, metabolism might adjust to compensate for changes in metabolite pools that occur during prolonged growth at low-temperatures, which would not occur in control seedlings germinated at 20°C.

A previous microarray study investigating seasonal flowering responses in wheat showed that transcript levels for key enzymes in the gibberellin biosynthesis pathway, ent-kaurene synthase and ent-kaurene oxidase, increase during step wise decreases in both temperature and photoperiod [46]. We found no evidence that these enzymes play a role in the vernalization response of barley seedlings; expression levels of *ENT-KAURENE SYNTHASE* (contig11470_at) and *ENT-KAURENE OXIDASE* (contig15315_at) remained at similar levels during and after vernalization (Figure 6K, L). The different findings of this study versus that of Winfield et al [46] might be due to the conditions used in each study; decreasing daylength versus darkness, or the age of plants examined (plants versus seedlings). Regardless, changes in transcript levels for these gibberellin biosynthetic enzymes are probably not required for the early stages of the vernalization response in barley seedlings. This does not rule out important roles for these genes in regulating responses to different temperature and daylength combinations, as suggested by Winfield et al [46], but highlights the advantage of using seedling vernalization as an experimental system; the effects of low-temperature can be separated from the effects of development or daylength. This is important because *sensu stricto* vernalization is a response to cold [16]–[19]. Furthermore, separating the effects of different seasonal flowering cues allows better prediction of physiological responses in complex environments.

Some contigs that show a rapid response to cold also show altered expression following prolonged cold treatment. For example, *COR14b* (HVSMEa0015E13r2_s_at) and *WCS19* (baak1p18_s_at) were induced by short term cold, similar to previous studies [34], [36], [43]. These genes were also activated by prolonged cold treatment and expression remained high in seedlings a day after prolonged cold treatment and in the leaves of vernalized plants (Figure 6G). This contrasts with the behaviour of most other cold responsive genes; *DHN5* for example, returns to expression levels similar to the control treatment when plants are shifted to warm temperatures after prolonged cold (Figure 6J). The COR14b and WCS19 proteins localise to the chloroplast, possibly to reduce photo-oxidative stress [52], [53], but it is unclear why expression of these contigs is maintained after cold treatment. The lasting change in expression of these contigs following prolonged exposure to cold could be mediated by changes in chromatin state. Cold induced histone modifications are maintained at the promoters of some cold responsive genes in *Arabidopsis thaliana*, although expression of these genes is not maintained [54].

Two contigs corresponding to *CBF* genes were expressed at lower levels in the leaves of vernalized plants; *CBF2* (AF442489_at) and *CBF9* (HVSMEn0019L21f_at) (Figure 6I, Table S9). Reduced expression of these contigs in the leaves of vernalized plants, which are beginning reproductive growth at the time point sampled [20], might contribute to the reduced capacity for cold acclimation that is associated with reproductive growth in cereals [3]. The equivalent *CBF* genes show elevated expression during cold acclimation in the *T. monococcum VRN1* deletion mutant (*mvp*), which is unable to initiate reproductive growth, consistent with this hypothesis [37]. It might be possible to increase frost tolerance during flowering if expression of these genes could be maintained during reproductive growth, through constitutive expression in transgenic plants for example. Increased frost tolerance during flowering would be a valuable trait in areas where sudden spring frosts occur at the time of flowering, causing reduced yield.

In conclusion, we have identified barley genes that respond to prolonged cold and show lasting changes in transcriptional activity when plants are shifted to normal growth conditions. The observation that only a limited number of contigs show lasting responses to prolonged cold, at least within the detection limits of microarray analysis, highlights the importance of *VRN1* in the vernalization response of temperate cereals. A key question for further research is how does prolonged cold lead to increased *VRN1* expression? By identifying genes that are differentially expressed during short and prolonged cold we have begun to address this question.

## Materials and Methods

### Plant Growth

To compare the effects of short and prolonged cold on transcription in barley seeds (cv. Sonja; a well characterised vernalization-responsive barley [20] with the genotype: *HvVRN1*, *HvVRN2*, *PPD-H1*, *ppd-H2*) were germinated and grown in darkness to an average coleoptile length of 4 cm at either 20°C over 5 days (control) or 4°C over 49 days (prolonged cold). Seedlings were then shifted from the control treatment to 4°C for 24 hours (short term cold) or shifted from the prolonged cold treatment to 20°C for 24 hours (post cold) (Figure 1A). In all treatments the shoot apex remained at an early stage of vegetative development, but plants grown from seedlings that experienced prolonged cold flower rapidly when shifted to normal growth temperatures, unlike control seedlings germinated at 20°C [20]. Seedlings were harvested from each treatment for RNA extraction.

To examine gene expression after prolonged cold, barley plants (*Hordeum vulgare*) (cv. Sonja) were grown in pots covered in foil at 4°C for 49 days. The foil was then removed and plants were grown in a glasshouse (18±2°C) in long days (16-h light/8-h dark), with supplementary light when natural levels dropped below 200 µE until they reached the three leaf stage (10 days) (Figure 1B). Non-treatment control plants were grown at the same time under the same conditions and were sampled at the equivalent stage of development (Figure 1B).

### Microarray analysis

RNA was extracted using the method of Chang et al. [55] and then further purified using RNeasy columns (Qiagen). Sample labelling and hybridisation to the Barley1 Gene chip [39] were conducted at the Australian Genome Research Facilities (AGRF; Melbourne, VIC, Australia), following the manufacturer's recommendations (Affymetrix, Santa Clara, CA). Microarray analyses were performed on 3 biological replicates of each sample. The resulting dataset was analysed in R v2.7.1 and analysed using packages from Bioconductor [56] (http://www.bioconductor.org/), with default settings. Normalisation was carried out by Robust Multichip Analysis (RMA) and differentially expressed contigs were identified across the normalised microarray datasets for biological replicates using linear modelling in limma in R v2.7.1 (Linear Models for Microarray Data) [57]. Each experimental sample was compared with the control sample (e.g. Short Cold vs Control), or to each other, and multiple testing was corrected for by controlling the FDR [58]. For PCA clustering, genes that were differentially expressed in any of the three seedling treatments relative to the control (empirical Bayes test, no minimum fold change cut off applied; 11057 contigs) were grouped based on condition using the 'cluster.samples' function in smida (R v.2.7.1). The method chosen was ‘pca’ and ‘euclidean’ was selected as the distance measured. The clusters were plotted using the first two principal components from the PCA analysis. Comparisons between lists of contigs with significantly changed expression in the different treatments and the generation of preliminary Venn diagrams was performed using the FiRe macro in Excel® [59]. Raw microarray data has been deposited in the Plant Expression Database (www.plexdb.org), a MIAME/Plant Compliant Gene Expression Resources for Plants and Plant Pathogens (Experiments BB94 and BB95).

### K-means Clustering

K-means cluster analysis was performed contigs that showed a two fold or greater change in transcript levels (p<0.01,) in any of the two-way comparisons between the different seedling treatments (1800 in total, Table S1, S2, S3, S4, S5, S6). Cluster analysis was performed using the MeV software from the TM4 microarray software suite using the default settings (Euclidean distance, and a maximum of 50 iterations), using the RMA normalized expression values from all 12 samples (3 replicates for each of the four treatments) [60]. Contigs that did not group with the primary clusters or that showed high variability between replicates were manually omitted from the final clusters (Table S11).

### Gene Expression Analysis

Total RNA was extracted using the method of Chang et al., [55] or the Qiagen RNeasy Plant Miniprep kit (Qiagen). cDNA was synthesised using an oligo(T) primer (T18[G/C/A]) to prime first-strand complementary DNA (cDNA) synthesis from 1-5 µg of total RNA with SuperScript III reverse transcriptase enzyme (Invitrogen). qRT-PCR was performed on a Rotor-Gene 3000 real-time cycler (Corbett Research). The primers used for *HvACTIN*, *HvVRN1 and HvOS2* have been described previously [47], [61]. The sequence of primers used for contig6358_at were as follows; forward 5′ TCCTCGTGTGATTTTGCAG 3′ and reverse 5′ TTGAGTTCAGCGATGCTACG 3′ and for HU14M19u_at; forward 5′ TCAAAAAGGATGCCCAAAAG 3′ and reverse 5′ AACAAGCTTGGCAAAACACA 3′. qRT-PCR was performed using Platinum Taq DNA polymerase (Invitrogen) with SYBR green. Cycling conditions were 4 minutes at 94°C, 40 cycles of 10 s at 95°C, 15 s at 60°C, and 20 s at 72°C, followed by a melting-curve program (72°C–95°C with a 5-s hold at each temperature). Fluorescence data were acquired at the 72°C step and during the melting-curve program. Expression levels of genes of interest were calculated relative to *ACTIN* using the comparative quantification analysis method (Rotogene-5; Corbett Research), which takes into account the amplification efficiency of each primer set. Data presented are the average of a minimum of three biological replicates (unless stated otherwise) and the error bars show standard error.

## Supporting Information

Figure S1 Quantitative RT-PCR assay versus array analysis of gene expression for selected contigs. A. Expression values as assayed by Affymetrix Barley1 chip, RMA normalisation, for four contigs with contrasting expression patterns and different activity levels. *HvVRN1* (rbaal14f06_s_at), a gene encoding a ribosomal protein (HU14M19u_at), a zinc finger transcription factor gene (contig6538_at), and *HvOS2* (contig12031_at). B. Quantitative RT-PCR assay of expression levels for the same contigs. Expression is shown relative to *ACTIN*. Error bars show standard error.
(TIF)

Table S1 Contigs with altered expression in the short cold treatment relative to no treatment control.
(XLS)

Table S2 Contigs with altered expression in the prolonged cold treatment relative to the no treatment control.
(XLS)

Table S3 Contigs with altered expression 1 day after prolonged cold treatment relative to the no treatment control.
(XLS)

Table S4 Contigs with altered expression in the prolonged cold treatment relative to the short cold treatment
(XLS)

Table S5 Contigs with altered expression after one day after prolonged cold relative to short cold treatment.
(XLS)

Table S6 Contigs with altered expression in the prolonged cold treatment relative to one day after prolonged cold treatment.
(XLS)

Table S7 Numbers and descriptions of contigs clustered together according to expression behaviour.
(XLS)

Table S8 Contigs with altered expression in the leaves of barley plants after vernalization.
(XLS)

Table S9 Contigs represented in primary cluster analysis with altered expression in the leaves of barley plants after vernalization.
(XLS)

Table S10 A core set of low-temperature responsive contigs in barley.
(XLS)

Table S11 Contigs omitted from top the ten main clusters.
(XLS)
