# Supplementary figures and images for: Transcriptome Analysis of the Vernalization Response in Barley (Hordeum vulgare) Seedlings
Source: PLoS One. 2011 Mar 9;6(3):e17900. doi: 10.1371/journal.pone.0017900 (PMC3052371; doi:10.1371/journal.pone.0017900)

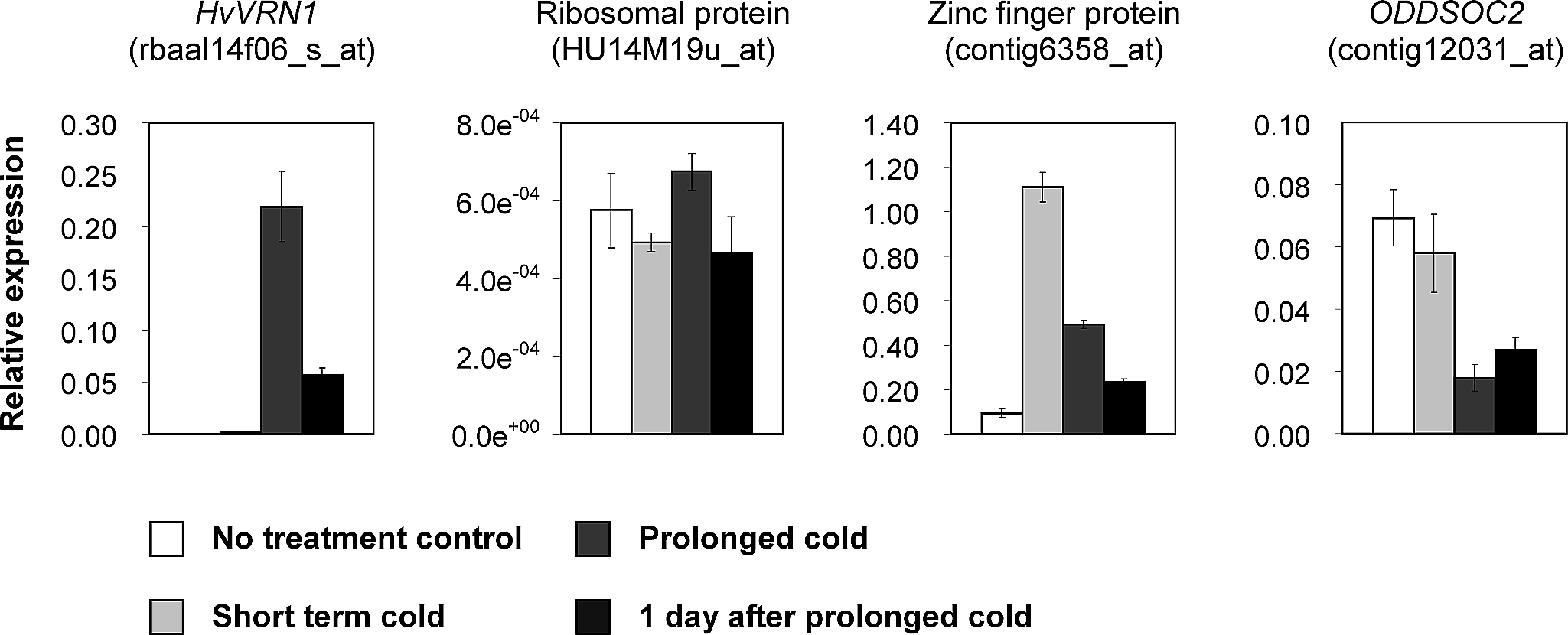

Supplement: Figure S1 — Quantitative RT-PCR assay versus array analysis of gene expression for selected contigs. A. Expression values as assayed by Affymetrix Barley1 chip, RMA normalisation, for four contigs with contrasting expression patterns and different activity levels. HvVRN1 (rbaal14f06_s_at), a gene encoding a ribosomal protein (HU14M19u_at), a zinc finger transcription factor gene (contig6538_at), and HvOS2 (contig12031_at). B. Quantitative RT-PCR assay of expression levels for the same contigs. Expression is shown relative to ACTIN. Error bars show standard error. (TIF) [file pone.0017900.s001.tif]
